# Recent Advances in Co_3_O_4_-Based Composites: Synthesis and Application in Combustion of Methane

**DOI:** 10.3390/nano13131917

**Published:** 2023-06-23

**Authors:** Xinfang Wei, Jiawei Kang, Lin Gan, Wei Wang, Lin Yang, Dijia Wang, Ruixia Zhong, Jian Qi

**Affiliations:** 1 Key Laboratory of Dielectric and Electrolyte Functional Material Hebei Province, Northeastern University at Qinhuangdao, Qinhuangdao 066004, China; kjw101610183@163.com (J.K.); ganlin0224xt@163.com (L.G.); 2172307@stu.neu.edu.cn (W.W.); yl20010508@yeah.net (L.Y.); dijia1218@163.com (D.W.); 2 State Key Laboratory of Biochemical Engineering, Institute of Process Engineering, Chinese Academy of Sciences, Beijing 100190, China; 3 School of Chemical Engineering, University of Chinese Academy of Sciences, Beijing 100049, China

**Keywords:** combustion of methane, nano-Co_3_O_4_, Co_3_O_4_-based composites

## Abstract

In recent years, it has been found that adjusting the organizational structure of Co_3_O_4_ through solid solution and other methods can effectively improve its catalytic performance for the oxidation of low concentration methane. Its catalytic activity is close to that of metal Pd, which is expected to replace costly noble metal catalysts. Therefore, the in-depth research on the mechanism and methods of Co_3_O_4_ microstructure regulation has very important academic value and economic benefits. In this paper, we reviewed the catalytic oxidation mechanism, microstructure regulation mechanism, and methods of nano-Co_3_O_4_ on methane gas, which provides reference for the development of high-activity Co_3_O_4_-based methane combustion catalysts. Through literature investigation, it is found that the surface energy state of nano-Co_3_O_4_ can be adjusted by loading of noble metals, resulting in the reduction of Co–O bond strength, thus accelerating the formation of reactive oxygen species chemical bonds, and improving its catalytic effect. Secondly, the use of metal oxides and non-metallic oxide carriers helps to disperse and stabilize cobalt ions, improve the structural elasticity of Co_3_O_4_, and ultimately improve its catalytic performance. In addition, the performance of the catalyst can be improved by adjusting the microstructure of the composite catalyst and optimizing the preparation process. In this review, we summarize the catalytic mechanism and microstructure regulation of nano-Co_3_O_4_ and its composite catalysts (embedded with noble metals or combined with metallic and nonmetallic oxides) for methane combustion. Notably, this review delves into the substance of measures that can be used to improve the catalytic performance of Co_3_O_4_, highlighting the constructive role of components in composite catalysts that can improve the catalytic capacity of Co_3_O_4_. Firstly, the research status of Co_3_O_4_ composite catalyst is reviewed in this paper. It is hoped that relevant researchers can get inspiration from this paper and develop high-activity Co_3_O_4_-based methane combustion catalyst.

## 1. Introduction

### 1.1. Background

In the current era of rapid technological advancement, coal has become an important energy source and plays a crucial role in industrial production and daily life. However, with the extensive extraction of coal, coal-bed gas tends to continuously dissipate from the coal seams, with a significant proportion (approximately 70%) of the gas being methane, which has a concentration between 0.1% and 1.0% of coal mine exhaust gas.

The direct discharge of methane, without any treatment, constitutes 23 times the quantum of carbon dioxide [1,2]. The harmful effects of high methane concentration on the environment are multifaceted, from its role as a greenhouse gas causing global warming to its potential to cause explosions. Moreover, the emissions of methane also have the potential to pollute the air and jeopardize human health. Consequently, reducing methane emissions has become crucial, and one of the most promising methods is to promote methane degradation.

The chemical process of C–H bond degradation needs to raise the temperature, which makes the low-temperature conversion of methane thermodynamically unfavorable. Therefore, in order to accelerate the catalytic oxidation of methane, there is an urgent need for an effective catalyst for low concentration methane oxidation [3].

### 1.2. Co_3_O_4_ Catalyst Materials

A large number of methane oxidation catalysts have been developed, including hexaaluminate [4], noble metal [5,6], perovskite [7], and transition metal oxides [8]. Owing to their cost-effectiveness, relative abundance, and high activity, transition metal oxides have come to the forefront of methane catalytic combustion research [9,10]. Among all single metal oxide catalysts, Co_3_O_4_ has emerged as one of the most significant catalyst materials, owing to its superior catalytic performance [11,12,13,14,15]. Especially, nano-Co_3_O_4_ catalysts have some advantages, including larger surface area, extremely rich pore structure, and higher reactivity. However, the actual reaction efficiency of these catalysts tends to plummet significantly under actual reaction conditions, owing to the instability of nano-Co_3_O_4_ during the reaction process, which leads to inactivation. In light of this, researchers have developed the Co_3_O_4_ composite doped with elements, which constitutes one of the most widely studied catalysts for methane combustion. Moreover, with the continuous exploration of scientific researchers, it has been established that composite materials loaded onto the support of Co_3_O_4_ can also improve the catalytic performance. The catalytic reactivity of cobalt ions can be enhanced by dispersing and stabilizing them. However, the synthetic methods and the use of different carriers in this process have important effects on the structure and texture properties. These various Co_3_O_4_ composite catalysts demonstrate significant potential in methane combustion, catering to the diverse applications of industrial production and social life, and promising great commercial value. Nevertheless, while there have been numerous reports on the synthetic methods of Co_3_O_4_ composites and their applications in methane combustion, most of these reports are presented separately, with no relevant overview of Co_3_O_4_ composites.

### 1.3. Contents of This Review

This paper attempts to fill this research gap by reviewing the recent research progress of Co_3_O_4_ composites. The applications of Co_3_O_4_ composite doped with elements and the composite loaded with Co_3_O_4_ on the carrier in methane combustion are discussed, focusing on the effect of the composites on Co–O bonding, followed by an analysis of the present challenges in the application of Co_3_O_4_.

## 2. Catalytic Oxidation of Methane by Nano-Co_3_O_4_

**Figure 1:**
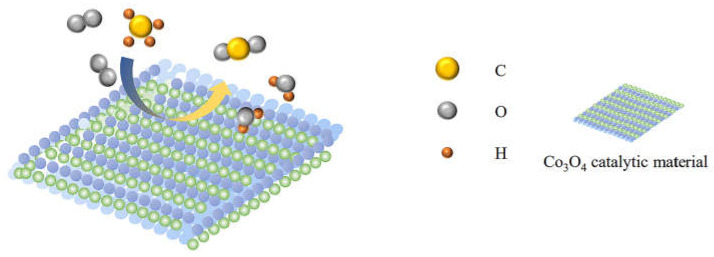
Description of methane oxidation catalyzed by Co_3_O_4_ catalytic material.

Co_3_O_4_, an exemplary metal oxide, manifests two kinds of cobalt oxides, namely CoO and Co_2_O_3_. This compound, akin to a typical cubic spinel structure, presents a face-centered cubic structure accumulation body, with Co^3+^ residing at the core of octahedron and CO^2+^ residing at the core of tetrahedron. Notably, Co_3_O_4_ has a feeble Co–O bond [16,17], resulting in the facile migration of surface oxygen vacancy [18]. In addition, Co_3_O_4_ exhibits a predisposition for oxygen vacancy production even at room temperature [3,19,20,21,22,23]. Furthermore, the 3D orbital of Co is not entirely filled, thereby accelerating the Co^2+^ ↔ Co^3+^ cycle [24,25]. As a result, the decomposition ability of oxygen and the migration rate of oxygen increase [26,27]; thus, Co_3_O_4_ has high activity in methane combustion compared with other single metal oxide catalysts. Consequently, Co_3_O_4_ has become a hot research topic in methane catalytic combustion [19,28,29,30]. Figure 1 depicts the process of catalytic methane combustion on the surface of the Co_3_O_4_ catalyst.

Currently, common models that describe the methane catalytic conversion are as follows:

(1) Eley–Rideal (E–R) model: In this model, oxygen molecules are adsorbed on the catalyst surface. Methane molecules react with oxygen adsorbed on the surface of the catalyst to form products such as CO, CO_2_, H_2_O, and formaldehyde and formic acid. The content of this process is as follows, where ※ represents the empty active site and X※ represents the adsorbed X.

O_2_ + 2※ → 2O※ (1)

2O※ + CH_4_ → HCHO※ + OH※ (2)

HCHO※ + O※ → CHO※ + OH※(3)

CHO※ + O※ → CO※ + OH※(4)

CO※ + O※ → CO_2_※ + ※ (5)

CO_2_※ → CO_2_ + ※ (6)

OH※ + OH※ → H_2_O※ + ※ (7)

H_2_O※ → H_2_O + ※ (8)

This model requires high O_2_ adsorption capacity, high oxygen migration capacity, and high electron affinity, and is commonly seen in catalysts containing metal oxides, such as CeO_2_, MnO_x_, Co_3_O_4_, Fe_2_O_3_, and NiO.

(2) Langmuir–Hinshelwood (L–H) model: In this model, methane and oxygen molecules are adsorbed on the catalyst surface and form active adsorbent species. These active adsorbent species react further to form products such as CO_2_, water, and formaldehyde. When the adsorbed methane reacts directly with oxygen, the reaction process can be divided into the following steps, where ※ represents the active adsorption site and X※ represents the X adsorbed on the active site.

CH_4_ + ※ → CH※ (9)

O_2_ + 2※ → 2O※ (10)

CH_4_※ + O※ → CH_3_※ + OH※ (11)

CH_3_※ + O※ → HCHO※ + H※ (12)

HCHO※ + O※ → CHO※ + H※(13)

CH※ + ※ → C※ + OH※(14)

C※ + O※ → CO※ + ※(15)

CO※ + O※ → CO_2_※ + ※ (16)

CO_2_※ → CO_2_ + ※ (17)

OH※ + OH※ → H_2_O※ + O※ (18)

H_2_O※ → H_2_O + ※ (19)

In this model, some metal catalysts, such as Pd, Rh, Pt, and other noble metal catalysts, are commonly used as L–H model catalysts in methane oxidation. In addition, some alkali metal oxide catalysts that promote the L–H model, such as Cs_2_O, Na_2_O, Li_2_O, etc., can also be used to catalyze methane oxidation.

(3) Mars–van Krevelen (MvK) model: In the MvK model, methane is first adsorbed on the surface of the catalyst and then reacts with lattice oxygen, which consists of gaseous oxygen in the feed gas, to form carbon dioxide and water. Like the L–H model, methane adsorbed can be converted into different intermediates. In this model, the oxygen content on the catalyst surface and the conversion between the catalyst oxide and the reduced substance are the key factors affecting the reaction.

(4) Two-term model: This model is also known as the mixing model. The catalytic combustion model of methane may vary with reaction conditions such as temperature and oxygen partial pressure. For example, Zasada et al. [31] found that the reaction model of Co_3_O_4_ changed from L–H to MvK with the change of reaction temperature.

Therefore, the model of catalytic oxidation of methane by Co_3_O_4_ can be reduced to the following three key steps:

(1) Methane and oxygen adsorption to Co_3_O_4_ catalytic material surface.
(2) The formation of active intermediate substances.
(3) Further reaction of active intermediate substance.

**Figure 2:**
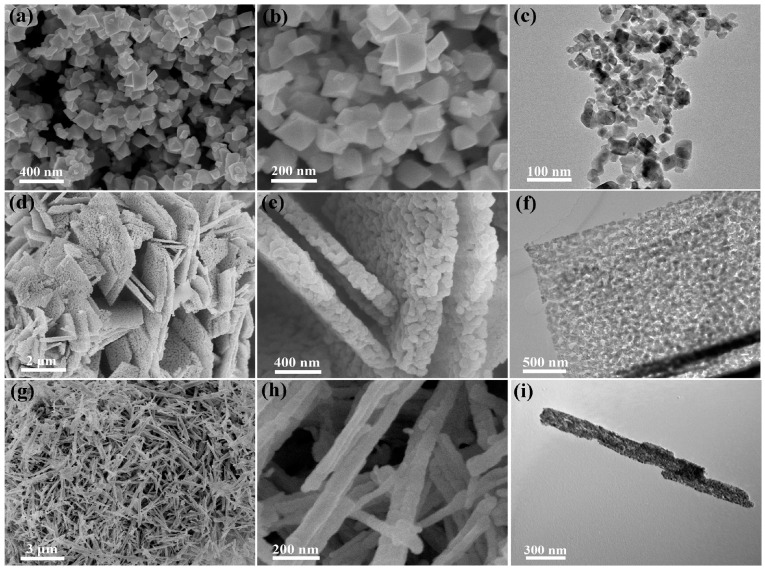
SEM and TEM images of (**a**–**c**) Co_3_O_4_-octahedrons, (**d**–**f**) Co_3_O_4_-plates, and (**g**–**i**) Co_3_O_4_-rods Reproduced with permission from Wang, K., Solvent-Free Chemical Approach to Synthesize Various Morphological Co_3_O_4_ for CO Oxidation; published by ACS Applied Materials & Interfaces 2017 [32].

Through an exceedingly facile solvent-free process [32], Co_3_O_4_-octahedron, -plate, and -rod nanomaterials were successfully synthesized, thereby augmenting the micromorphology of Co_3_O_4_ nano-materials as characterized by SEM and TEM. In Figure 2a–c, the shape of as-obtained Co_3_O_4_ samples demonstrated the quintessential octahedron-like configuration, with a size ranging from 100 to 200 nm. Meanwhile, Co_3_O_4_-plate nanomaterials were composed of a jumbled mass of closely packed irregular particles, with a size of 70–80 nm. Remarkably, the plate-like Co_3_O_4_ nanomaterials exhibited a plethora of well-defined pore structures, as clearly depicted in Figure 2d–f. In contrast, the synthesis of Co_3_O_4_-rods with a diameter of approximately 50 nm and a length of several micrometers was achieved through the facile thermal decomposition of the precursor, CoC_2_O_4_·2H_2_O, obtained under mild conditions. As evidenced by Figure 2g–i, the surface pore structures of rod-like Co_3_O_4_ nanomaterials were clearly discernible, which could be attributed to the sequential liberation of the gas emanating from the decomposition of the precursor during calcination.

The catalytic process is a highly complex phenomenon that involves a series of intricate steps, including adsorption, decomposition, continuous decomposition, and desorption processes occurring on the surface of the catalyst. As a result, the catalytic effect of a given process is strongly dependent on the size and surface morphology of the catalyst particles, making the design and preparation of catalysts with appropriate morphology and size a key concern in the field of catalyst research [33,34].

**Figure 3:**
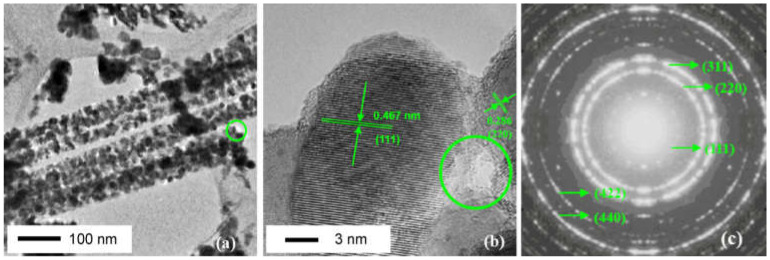
HTEM (high transmission electron microscopy) micrographs of the porous Co_3_O_4_ nanorods; (**a**) TEM (transmission electron microscopy) image; (**b**) lattice spacing image; (**c**) ED patterns. Reproduced with permission from F. Teng, High combustion activity of CH_4_ and catalluminescence properties of CO oxidation over porous Co_3_O_4_ nanorods; published by Applied Catalysis B-Environmental, 2011 [48].

Throughout history, researchers have been working tirelessly to synthesize Co_3_O_4_ with varying morphologies [35,36,37,38], ranging from cubes [39] and sheets [40] to petals [41], lines, tubular structures [42], spherical particles [43], and even nano rod [44]. Each of these morphologies exposes a different crystal surface, which in turn affects the catalytic activity of the resulting material. For example, Co_3_O_4_ nanorods exhibit the highest catalytic activity, because of the large number of exposed, highly active (112) crystal surfaces [45,46]. In contrast, the catalytic activity of nanoparticle catalysts with a (001) surface is comparatively low [47]. Fei et al. [48] used hydrothermal method to make the prepared Co_3_O_4_ nanorods have higher specific surface area and richer pore size. The resulting material exhibits excellent catalytic activity in the catalytic combustion of methane, thanks in part to its large surface area and abundant pore structure. Polycrystalline properties of Co_3_O_4_ nanorods can be confirmed using a variety of analytical techniques. For example, Figure 3 shows the polycrystalline structures of Co_3_O_4_ nanorods, which are composed of nanoparticles measuring approximately 10 nm. As can be seen from Figure 3a, the contrast between light and dark regions confirms that the nanoparticles do not form a solid whole. At higher magnification, the porosity of Co_3_O_4_ nanorods becomes even more evident. Figure 3b shows a hole measuring approximately 4 nm, which can be observed through the contrast between light and dark regions. The lattice fringe spacing is 0.467 nm and 0.286 nm, corresponding to the (111) and (220) planes of Co_3_O_4_ crystal, respectively. Finally, Figure 3c shows the ED (electron diffraction) pattern of the sample, calculated by analyzing the corresponding spacing and planes (111), (220), (311), (422), and (440). Despite the many advantages of Co_3_O_4_ nanotubes with (112) crystal surface, their poor stability and limited catalytic activity under high temperatures have been a significant concern. To address this issue, researchers have developed modified Kirkendall effect methods for synthesizing Co_3_O_4_ nanotubes with better thermal stability and higher catalytic activity. Fei et al. [42] synthesized Co_3_O_4_ nanotubes with the morphology guidance of modified Kirkendall effect, and analyzed that the main exposed (112) crystal surface showed good catalytic activity and stability below 700 °C. However, in the actual use process, the temperature in the methane reactor is uncontrollable. When the working environment temperature is too high, nano Co_3_O_4_ will be sintered and the catalyst will be inactivated, so the stability is poor and the catalytic activity cannot be guaranteed [49]. Prasad. et al. [50] added 4.5% CO to the gas during calcination, ultimately resulting in a sample with the largest specific surface area, the smallest nanocrystalline, and a highly dispersed anoxic structure defect morphology. Compared to traditional Co_3_O_4_, this new material exhibited a 40–80 °C reduction in catalytic temperature. Andoni Choya et al. [51] prepared bulk pure Co_3_O_4_ by eight methods for lean methane combustion, where basic precipitation employing sodium carbonate as the precipitating agent showed the best catalytic capacity. The reasons for this can be attributed to the following two factors: firstly, there is lattice distortion in the samples made by this method, which leads to more abundant oxygen vacancies, and secondly, the sample surface has the highest Co^3+^ abundance, which in turn leads to an increase in lattice oxygen abundance. Table 1 lists the surface area and the corresponding preparation temperature of nanometer Co_3_O_4_ mentioned above.

## 3. Co_3_O_4_ Composite Catalysts in Combustion of Methane

**Figure 4:**
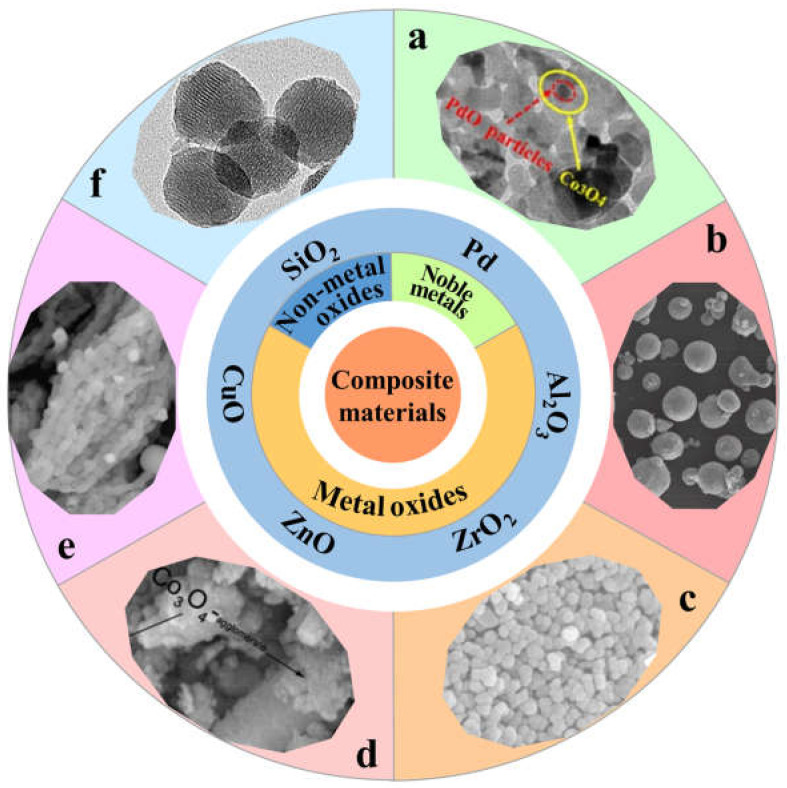
Several common composite materials: (**a**) Pd, reproduced with permission from ref. [82], Copyright 2021, Applied Surface Science; (**b**) Al_2_O_3_, reprinted with permission from ref. [63], Copyright 2020, Elsevier; (**c**) ZrO_2_, reprinted with permission from ref. [65], Copyright 2017, Elsevier; (**d**) ZnO, reprinted with permission from ref. [66], Copyright 2013, Wiley-VCH; (**e**) CuO, reprinted with permission from ref. [68], Copyright 2014, Springer; (**f**) SiO_2_, reprinted with permission from ref. [83], Copyright 2012, Elsevier.

In the quest to augment the thermal stability of Co_3_O_4_ in the catalytic process and elevate the catalyst’s catalytic activity in the combustion of methane [52], researchers customarily introduce doping of other elements in the preparation of Co_3_O_4_ or heap it on other supports [33,53,54,55,56,57]. The utilization of carriers can scatter and stabilize cobalt ions and hasten the Co^2+^ ↔ Co^3+^ reaction [58]. While assembling the catalyst, distinct preparation techniques can potentially impact the specific surface area, micro morphology, the degree of dispersion of the active phase of the catalyst, and other structural regulations, which can ultimately influence the activity of the catalyst [59,60,61]. There are three common methods to improve the catalytic performance of Co_3_O_4_ catalysts (as shown in Figure 4): (1) noble metal doping; (2) compounding with metal oxides (Al_2_O_3_ [62,63,64], ZrO_2_ [65], SnO_2_, ZnO [66], and CuO [67,68,69] are common); and (3) loading onto non-metal oxides (SiO_2_, porous ceramics [70]). The strength of Co–O bond can be weakened by combining with noble metals [71,72,73], which can increase the mobility of oxygen and promote the formation of active oxygen, so as to improve its catalytic effect [50,74,75,76,77,78,79,80,81]. In the subsequent sections, we will explain noble metals, metal oxides, and non-metallic oxides. Table 2 describes the action mechanism of three measures to enhance the catalytic performance of Co_3_O_4_ catalyst for methane oxidation catalyzed by Co_3_O_4_.

### 3.1. Noble Metals

In the process of oxidizing volatile organic compounds and CO, noble metals have shown great potential as active substances. However, due to their high price, researchers are currently focusing on finding an optimal doping ratio with other substances to reduce costs. When noble metal ions are introduced into the lattice of Co_3_O_4_ in doped composites, they have the ability to inhibit electrons and holes from forming trapping centers in the compound. The doping of noble metals mainly affects the Co–O bond in the following two ways. First, the doping of noble metals can introduce more oxygen vacancies and increase the Co–O bond content on the surface of Co_3_O_4_, improving the activity and stability of the catalyst. In addition, in the catalytic reaction process, the doping of noble metals can also affect the fracture and formation of Co–O bond, promote the formation and release of Co_3_O_4_ surface oxygen species in the methane oxidation reaction, and then promote the catalytic reaction. As a result, impurity levels are often formed, which can cause lattice defects and form more active sites, leading to an improved catalytic oxidation performance of Co_3_O_4_. To obtain superior performance catalyst, researchers often dope Pd, Au, Ag, Pt [84], and other elements with Co_3_O_4_ to obtain superior performance catalyst.

Palladium (Pd), a well-known noble metal, has earned an excellent reputation for its ubiquitous use in catalysts [84,85,86]. The empirical evidence suggests that Pd not only performs exceedingly well, but it also reigns supreme as the most active material in combustion. In fact, a myriad of methane studies has been conducted, and all have demonstrated Pd catalyst’s remarkable combustion potential. However, the catalytic performance of Pd-based catalysts is a complex and multifaceted phenomenon, with numerous factors affecting their efficacy. These factors include the valence of the catalyst’s constituent elements, the dispersion of these elements, and the support properties [87,88,89,90,91,92,93,94,95,96].

The synergistic interaction between the metal oxide and mixed metal oxide matrix is another crucial determinant of Pd catalyst’s catalytic performance. It is worth noting that different morphologies of Co_3_O_4_ and its composites can produce divergent catalytic effects owing to the diverse crystal planes exposed by Co_3_O_4_. Moreover, the catalytic activity of Pd combined with various morphologies of Co is substantially higher in the catalytic process than that of Pd and directly prepared Co_3_O_4_ [97,98]. This catalytic activity is especially noticeable in Pd combined with petal-shaped, rectangular-shaped, and cubic Co_3_O_4_, where the catalytic potential is markedly superior to that of Pd and directly prepared Co_3_O_4_.

**Figure 5:**
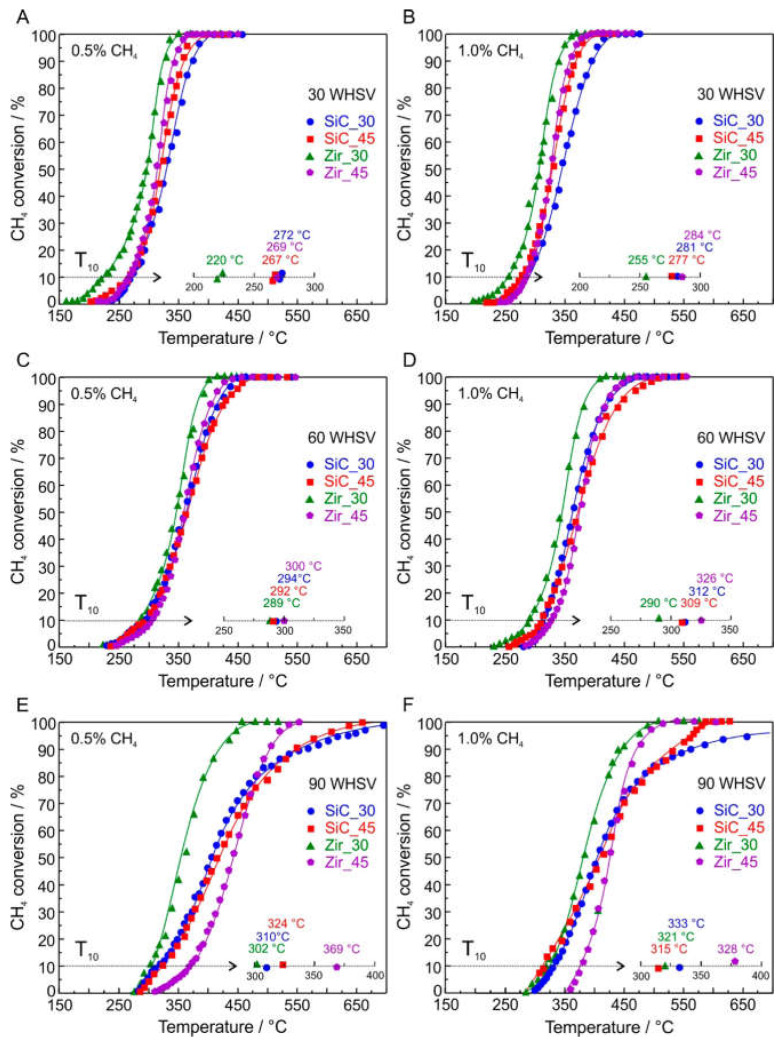
CH_4_-TPO (temperature programmed oxidation) for all the coated OCF at various methane inlet concentrations and WHSV. In the inserts: comparison of the T_10_. (**A**) 0.5%CH_4_ and 30 WHSV; (**B**) 1%CH_4_ and 30 WHSV; (**C**) 0.5%CH_4_ and 60 WHSV; (**D**) 1%CH_4_ and 60 WHSV; (**E**) 0.5%CH_4_ and 90 WHSV; (**F**) 1%CH_4_ and 90 WHSV. Reproduced with permission from Ercolino, G, Catalytic Performance of Pd/Co_3_O_4_ on SiC and ZrO_2_ Open Cell Foams for Process Intensification of Methane Combustion in Lean Conditions; published by Industrial & Engineering Chemistry Research 2017 [93].

Ercolino et al. [91] delved into the intricate world of catalyst synthesis by implementing a solution combustion synthesis, combined with a humidification impregnation method to synthesize a Pd doped cobalt spinel catalyst. The addition of Pd proved to be a masterstroke, inducing the formation of a reduced cobalt oxide phase, which helped generate active oxygen, facilitating the complete oxidation of CH_4_ under conditions lower than 430 °C. The positive impact of Pd was primarily due to the existence of well-dispersed catalytic sites, which further accentuated the catalytic potential. In another study by Ercolino et al. [93], silicon carbide (SiC) and zirconia (Zir) open cells foams (OCF) with different pore per inch (ppi) density were coated with 200 mg of Co_3_O_4_ by solution combustion synthesis and doped with 3 wt.% of Pd via wetness impregnation. As shown in Figure 5, the catalytic activity of lean oxidation of methane was tested at different weight hour space velocities (WHSV). The results showed that the catalytic performance of SiC OCF was comparatively worse than that of Zir OCF, especially at high temperatures (close to T_90_). However, SiC OFC exhibited similar performance to Zir OCF at low temperatures and increased WHSV. The best catalytic activity was observed in Zir OCF with 30 ppi, followed by Zir 45 ppi, SiC 45 ppi, and SiC 30 ppi under various reaction conditions. Notably, the superior performance of Zir OCFs was attributed to their low total heat transfer coefficient, which aided in heat dissipation through flue gas convection. These results unequivocally highlight the pivotal role of thermal conductivity in the structural catalyst support.

**Figure 6:**
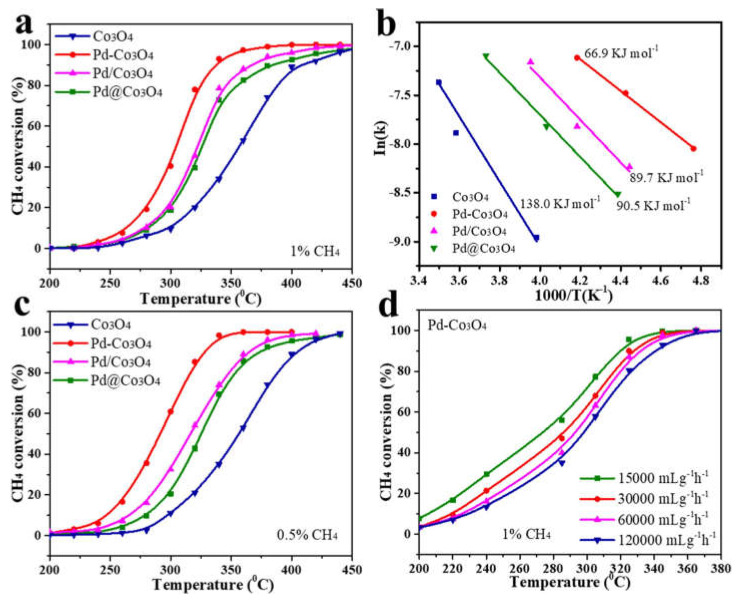
(**a**) CH_4_ conversion as a function of temperature under 1% CH_4_, (**b**) the corresponding Arrhenius plots of CH_4_ oxidation rates under 1% CH_4_, (**c**) CH_4_ conversion as a function of temperature under 0.5% CH_4_, and (**d**) CH_4_ conversion as a function of temperature with different WHSV under 1% CH_4_. Reproduced with permission from J. Xiong, The effect of existence states of PdO_x_ supported by Co_3_O_4_ nanoplatelets on catalytic oxidation of methane; published by Applied Surface Science, 2021 [82].

Xiong et al. [82] synthesized three kinds of nano-flake Co_3_O_4_-supported palladium catalysts to investigate the effects of palladium presence on methane catalytic combustion. They are Pd@Co_3_O_4_ (Pd as the core and Co_3_O_4_ as the shell), Pd/Co_3_O_4_ (Pd loaded on Co_3_O_4_), and Pd-Co_3_O_4_ (Pd doped with Co_3_O_4_). The sizes of the Co_3_O_4_ particles in all samples were determined using the Scherrer equation (Table 3), and it was found that loading Pd did not affect the crystal structure of Co_3_O_4_. Interestingly, the average grain size of Co_3_O_4_ in Pd-based samples remained similar to that of original Co_3_O_4_. The researchers then investigated the morphology and crystal structure of the catalyst using TEM and HRTEM (high resolution transmission electron microscopy), respectively. The Co_3_O_4_ carrier was found to be a two-dimensional nanosheet with a uniform ultra-thin structure, and after Pd embedding Co_3_O_4_, the microstructure of Pd-Co_3_O_4_ catalyst hardly changed. The investigation of Pd@Co_3_O_4_ catalyst has revealed some intriguing findings regarding its dispersion and combustion performance. Through the utilization of EDS (energy dispersive spectroscopy) element mapping, it was observed that PdO_x_ species were distributed uniformly on Pd-Co_3_O_4_ vectors while PdO_x_ nanoparticles were sparsely dispersed on the Co_3_O_4_ surface, indicating that PdO_x_ was firmly embedded in the Co_3_O_4_ lattice and displayed high dispersion. HRTEM images of Pd@Co_3_O_4_ catalyst further confirmed an interface between Co_3_O_4_ and PdO_x_, suggesting that PdO_x_ was enveloped by Co_3_O_4_. Moreover, EDS images displayed that the surface of PdO_x_ particles was covered by a thin, uniform layer of Co_3_O_4_, which corroborated the core-shell traits of Pd@Co_3_O_4_. Three cobalt-based catalysts were successfully prepared, and the combustion performance of Pd-Co_3_O_4_ catalyst for CH_4_ was compared with that of Pd/Co_3_O_4_ and Pd@Co_3_O_4_. The experimental results indicated that the Pd-Co_3_O_4_ catalyst exhibited superior catalytic activity and lower apparent activation energy than the other two catalysts. Furthermore, the Pd-Co_3_O_4_ catalyst was found to be highly resistant to the co-toxicity of H_2_O and CO_2_. This was due to the high Pd dispersion and the active adsorbed oxygen molecules and oxygen vacancies provided by the embedded Pd-Co_3_O_4_ structure, which resulted in better reducibility. Interestingly, the Pd-Co_3_O_4_ catalyst, which was prepared via a hydrothermal method, showed the highest catalytic effect on CH_4_ combustion, owing to the higher surface Pd^2+^ active substances. The apparent activation energy for the combustion reaction of CH_4_ followed the order of Co_3_O_4_ (138.0 kJ mol^−1^) > Pd@Co_3_O_4_ (90.5 kJ mol^−1^) > Pd/Co_3_O_4_ (89.7 kJ mol^−1^) > Pd-Co_3_O_4_ (66.9 kJ mol^−1^), as calculated according to the slope of the Arrhenius curve in Figure 6b. At a CH_4_ concentration of 0.5%, the Pd-Co_3_O_4_ catalyst still exhibited the highest catalytic activity (T_90_ = 326 °C), as shown in Figure 4c. The Pd-Co_3_O_4_ catalyst also demonstrated better CH_4_ oxidation activity at lower CH_4_ concentrations, as can be seen from Figure 6a,c. This was attributed to the possibility that the number of reactant molecules may exceed the oxidation capacity of surface reactive oxygen species at 1.0% CH_4_. The effect of WHSV on the catalytic performance of Pd-Co_3_O_4_ was also investigated, and it was found that methane conversion decreased slightly as the WHSV value increased from 15,000 to 120,000 mL g^−1^ h^−1^. The richer the surface Pd^2+^ active substances are, the more types and contents of surface adsorbed oxygen will be. This in turn affects the catalytic efficiency of methane, which is related to the migration rate and reducibility of lattice oxygen, both of which depend on the type and content of surface adsorbed oxygen and oxygen defects. Overall, the findings of this study suggest that the Pd-Co_3_O_4_ catalyst has great potential for oxidizing CH_4_ under the conditions of dilute combustion and high space velocity. The high dispersion of Pd and the unique embedded Pd-Co_3_O_4_ structure provide it.

Liotta et al. [99] prepared a powdered Pd/Co_3_O_4_ catalyst containing a small amount (0.7 wt.%) of noble metal for methane oxidation at the stoichiometric ratio of CH_4_/O_2_. However, the catalyst’s efficacy is significantly compromised by exposure to sulfur dioxide (SO_2_), prompting Liotta et al. to investigate the impact of SO_2_ on the activity of Co_3_O_4_ and supported Pd catalyst. To gain a deeper understanding of the deactivation process, X-ray photoelectron spectroscopy (XPS) analysis was performed on both Pd/Co_3_O_4_ and Co_3_O_4_ after the catalytic test. The resulting data, summarized in Table 4, reveal that the amount of surface palladium oxide was reduced by a factor of 10 after 4 SO_2_-free catalytic runs. This reduction in surface palladium oxide was accompanied by a corresponding decrease in activity, suggesting that catalyst deactivation may be linked to the sintering or diffusion of PdO_2_ in the absence of SO_2_. When comparing the results of four cycles without SO_2_ and with SO_2_, Liotta et al. observed a further reduction in the palladium content on the catalyst surface. Specifically, the Pd (at%) decreased from 0.4 to 0.2. The exposure of Pd/ Co_3_O_4_ to SO_2_ was found to induce palladium reduction under stoichiometric conditions, and Table 4 reports XPS data for samples treated with one and three successive sulfur-free tests. The sulfur content gradually decreased from 5.0 to 1.1 at %, resulting in a partial recovery of catalytic activity. Interestingly, the temperature at which methane was converted to 50% was transferred from 562 °C in the first run to 495 °C in the third run. In conclusion, Liotta et al. have developed a Pd/Co_3_O_4_ catalyst with a low noble metal content for methane oxidation. However, this catalyst is susceptible to deactivation by exposure to sulfur dioxide, which may lead to the sintering or diffusion of PdO_2_. Ongoing research is focused on developing regeneration treatments under reduction and/or oxidation conditions to mitigate the impact of SO_2_ poisoning on catalyst performance.

Indubitably, the chemical element gold has been subject to rather scant attention in the realm of catalysis, owing to its characteristics of inertness and low melting point. Nonetheless, since 1987, when certain intrepid researchers succeeded in producing a series of gold catalysts via the coprecipitation method, the oxidation of Co, and indeed, the catalytic activities of gold catalysts at low temperatures, have been shown to be strikingly vigorous and active, attracting an ever-increasing degree of notice and commendation in the scientific community. Reports of gold (Au) catalysts that display significant catalytic activity have proliferated, with various experiments investigating and exploring the capabilities of such catalysts in this domain. In spite of the considerable progress achieved with Au co-precipitated oxides in promoting total oxidation with the aid of several transition metals, the potential of gold as a catalyst in the combustion of methane remains relatively unexplored and underappreciated.

In an intrepid and innovative experiment, Miao et al. [100] employed the co-precipitation method to synthesize an Au-Pt/Co_3_O_4_ catalyst with the aim of facilitating methane combustion at a low temperature. This pursuit was accompanied by a meticulous optimization of the preparation process for the Au-Pt/Co_3_O_4_ catalyst, yielding some intriguing results. Specifically, the researchers discovered that the introduction of platinum into the mix notably enhanced the combustion activity, a phenomenon that can be mainly attributed to the compelling synergistic action between platinum and gold. This potent combination ultimately led to a significant improvement in the combustion activity of methane, thereby lending further credence to the use of such catalysts in this context. Moreover, the researchers made an additional noteworthy discovery, namely that the activity of Pd in the methane oxidation reaction surpassed that of Pt. However, this particular finding did not undermine the optimal loading ratio between Au and Pt, which remained the key to the effective catalytic combustion of methane.

**Figure 7:**
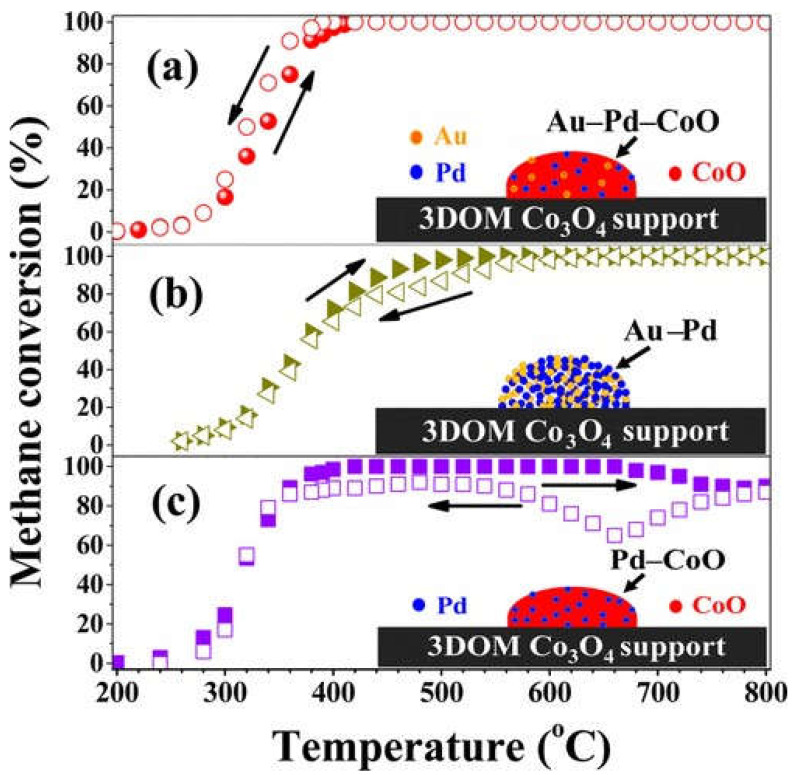
Methane conversion as a function of the temperature when the reaction temperature increased (solid) or decreased (open) over 3DOM Co_3_O_4_-supported (**a**) Au−Pd−3.61CoO, (**b**) Au−Pd, and (**c**) Pd−3.61CoO at SV = 20,000 mL g^−1^ h^−1^. Reproduced with permission from Shaohua Xie, Efficient Removal of Methane over Cobalt-Monoxide-Doped AuPd Nanocatalysts; published by Environmental Science & Technology 2017 [101].

Xie et al. [101] ventured to introduce a specific quantity of CoO into loaded Au–Pd alloy nanoparticles (NPs), an approach that led to the production of an exceedingly potent Au-Pd-xCoO/three-dimensionally ordered microporous (3DOM) Co_3_O_4_ (where x denotes the Co/Pd molar ratio) catalyst. Their findings demonstrated that the incorporation of CoO played a pivotal role in inducing the formation of the highly active PdO–CoO active site, which in turn facilitated the adsorption and activation of CH_4_ and ultimately resulted in an improvement in catalytic performance. It should be mentioned that Figure 7 revealed that the activity of the Au-Pd-3.61CoO/3DOM Co_3_O_4_ catalyst remained remarkably stable throughout the entire temperature range (400~800 °C), indicating that it was a highly durable catalyst for the oxidation of CH_4_. Interestingly, the methane conversion rate declined with increasing temperature, whereas it rose with decreasing temperature (300~400 °C) under the auspices of the Au-Pd-3.61CoO/3DOM Co_3_O_4_ catalyst. Furthermore, the researchers observed that the deactivation of 3DOM Co_3_O_4_-supported Au-Pd, Pd-CoO, and Au-Pd-xCoO nano-catalysts with water vapor was a direct result of the formation and accumulation of hydroxyl groups on the catalyst surface. Notably, the deactivation of the Pd-CoO/3DOM Co_3_O_4_ catalyst at high temperature (680~800 °C) was potentially attributable to the decomposition of the PdO_y_ active phase into clustered PdO NPs. Finally, it was revealed that the Au-Pd-xCoO/3DOM Co_3_O_4_ nano-catalysts boasted superior thermal stability and water resistance in comparison to Au-Pd and Pd-CoO nano-catalysts supported by 3DOM Co_3_O_4_.

In conclusion, doping of noble metals can enhance the oxygen activity and stability of Co–O bond and affect the fracture and formation of Co–O bond, thus raising the concentration of oxygen vacancies [102,103,104]. For instance, doping noble metals such as Pt and Pd can effectively introduce numerous oxygen vacancies while also boosting the Co–O bond content on composite catalyst material surfaces. In addition, appropriate doping of noble metals such as Au and Ag can also improve the strength of Co–O and promote the formation of Co–O bonds. Of course, it is possible to dope in multiple noble metals simultaneously to further enhance catalytic performance.

### 3.2. Metal Oxides

Metal oxides, as their name implies, are compounds formed by the combination of metal elements with oxygen. These metal oxides can be found for all metals, including the widely studied platinum (Pt) and gold (Au) metals. Consequently, doping metal oxides has been recognized as an essential research focus to explore the effects of doping on Co_3_O_4_ catalytic performance. Among various types of catalyst carriers, transition metal oxides play a vital role in catalytic reactions, particularly as transition noble metal catalysts for activating substitute hydrocarbons. Doped metal oxides can introduce oxygen vacancies into the Co_3_O_4_ lattice, forming more oxygen active sites on the Co–O bond [105], thereby increasing the oxygen reduction capacity of the Co_3_O_4_ catalyst surface. At the same time, this doping process can effectively improve the interaction force and coordination environment between Co and O ions in Co_3_O_4_ catalyst, promote the formation and stability of Co–O bond, and then improve the catalytic efficiency of methane oxidation reaction. As a result, the catalytic properties of composites containing metal oxides and Co_3_O_4_ have been extensively studied in recent years, making it an intriguing and exciting research topic in the field of catalysis.

Based on the study of Pd/Co_3_O_4_ catalysts and Au/Co_3_O_4_ catalysts, Yan et al. [106] synthesized Au@PdO_x_/Co_3_O_4_ nanorods with the epitaxial connection between the Au@PdO_x_ and Co_3_O_4_ domains through embedding Pd and Au seeds on the cobalt hydroxycarbonate precursor with further hydrothermal treatment and calcination. The excellent catalytic performance of the nanorods was found to be due to the structural effect between the PdO_x_-rich shell and Au-rich core, as well as the strong interaction of Au, Pd, and Co_3_O_4_, which benefits the activation of methane and enhances sintering and poison resistance required for practical applications. Table 5 presents a clear comparison of the performance of several catalysts. Based on T_10_ and T_90_, the methane combustion activity of the catalysts was found to be Au@PdO_x_/Co_3_O_4_ > Pd/Co_3_O_4_ > Au/Co_3_O_4_ > Co_3_O_4_. Moreover, the apparent activation energy of Au@PdO_x_/Co_3_O_4_ nanorods was observed to be approximately 50.9 kJ mol^−1^, which was lower than that of Pd/Co_3_O_4_ nanorods (63.0 kJ mol^−1^), Au/Co_3_O_4_ nanorods (53.5 kJ mol^−1^), and Co_3_O_4_ nanorods (74.0 kJ mol^−1^). These results indicate that the low temperature activation energy of Au/Co_3_O_4_ nanorods is higher than that of Pd/Co_3_O_4_ nanorods, suggesting that Au and Pd of Au@PdO_x_/Co_3_O_4_ catalyst have a positive synergistic effect on methane catalyzed combustion.

**Figure 8:**
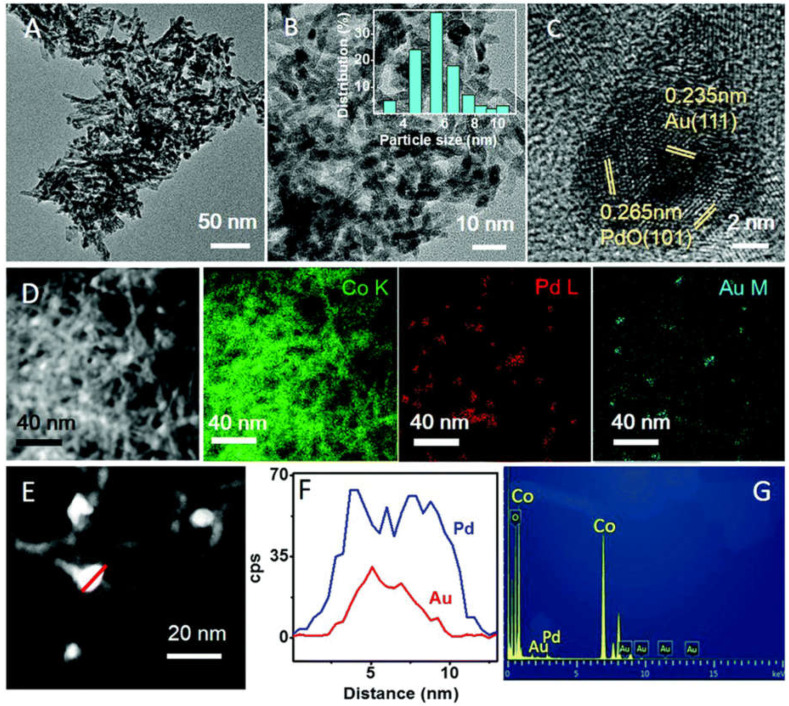
The morphology of the Au@PdO_x_/Co_3_O_4_: (**A**–**C**) TEM and HRTEM images, the insert in (**B**) is the size distribution of bimetal nanoparticles, (**D**) HAADF-STEM and EDS elemental mapping images, (**E**,**F**) HAADF-STEM and the line scanning profiles of a nanoparticle, and (**G**) the corresponding EDS spectra. Reproduced with permission from N. Yang, Au@PdO_x_ with a PdO_x_-rich shell and Au-rich core embedded in Co_3_O_4_ nanorods for catalytic combustion of methane; published by Nanoscale, 2017 [106].

The structure of the catalyst was studied by HRTEM, HAADF-STEM, and EDS mapping, which confirmed that the nanoparticles were anchored to the Co_3_O_4_ rod (Figure 8). As shown in Figure 8C, d values of 0.265 nm and 0.215 nm in the outer layer correspond to plane (101) and plane (110) of PdO, respectively, while d values of 0.235 nm in the center relate to plane (111) of Au. In the element mapping image shown in Figure 8D, most nanoparticles contain both Pd and Au. HAADF-STEM images and corresponding EDS line-scan spectra show that typical bimetallic nanoparticles are seen as rich in PdO shells and Au cores. According to EDS results (Figure 8G), the atomic ratio of Pd to Au on the surface was 11.6:1, higher than the atomic ratio of the bulk calculated by ICP-AES (inductively coupled plasma atomic emission spectra) results (Table 5) (9.3:1), which further confirmed that the nanoparticle had a new structure rich in PdO shell and Au core, so the nanoparticle was recorded as Au@PdO_x_.

In the study conducted by Hao et al. [49], a Co_3_O_4_–SnO_2_ hybrid oxide was synthesized using the coprecipitation method. The hybrid oxide displayed impressive catalytic activity and thermal stability during practical applications. The interaction between cobalt oxide and tin oxide was found to increase the oxygen migration efficiency through H_2_-temperature programmed reduction analysis. The dispersion of cobalt also contributed to its stability.

Wang et al. [107] prepared γ-Al_2_O_3_/Co_3_O_4_ (an alumina molecular sieve loaded on Co_3_O_4_) by combining initial wet impregnation and subsequent combustion synthesis. The catalyst has rich content of Co^3+^ and large dispersion [108], so the catalytic effect is good to fully oxidize methane at 350 °C. Zavyalova et al. [59] explored the use of various nanosized γ-Al_2_O_3_/Co_3_O_4_ catalysts with a defective structure that were synthesized via wetness impregnation. These catalysts exhibited remarkable activity in the total oxidation of methane.

CeO_2_/Co_3_O_4_ composites have proven to be highly effective for the combustion of methane [109,110,111,112,113,114,115]. However, during the catalytic combustion process, the production of SO_2_ can inhibit the reaction and reduce the reactivity of the catalyst [37]. This is where CeO_2_ comes in as a sort of SO_2_ scavenger. By forming cerium sulfate, CeO_2_ can provide superior SO_2_ tolerance to the catalyst [58,116,117,118,119,120]. Zeng et al. [120] used the microemulsion method to prepare CeO_2_/Co_3_O_4_ catalysts. They found that using a low precursor concentration when preparing the Co_3_O_4_ support can increase the specific surface area and result in smaller Co_3_O_4_ grain size. Liotta et al. [121] also developed a Co_3_O_4_-CeO_2_ bimetallic composite oxide on a cordierite honeycomb carrier. By adding a small amount of Pd-Pt metal to Co_3_O_4_-CeO_2_ in the working range of 400~600 °C, they were able to strongly enhance the oxidation activity of CH_4_ without any observed deactivation after five catalytic runs. In another experiment, Liotta prepared catalytic materials using the co-precipitation method. They observed a stabilizing effect due to the presence of ceria or ceria-zirconia against Co_3_O_4_ decomposition into CoO. Furthermore, the role of ceria was to disperse the active phase Co_3_O_4_ and promote reduction at low temperatures, maintaining good combustion activity of the cobalt composite oxides [122]. Dou et al. [123] prepared Co_3_O_4_/CeO_2_ nanocomposite catalysts with Co_3_O_4_ nanoparticles as the carrier and cerium dioxide nanorods as the active phase using the deposition precipitation method. Their catalytic activity was found to be higher than that of pure Co_3_O_4_ and CeO_2_, and they exhibited excellent catalytic activity at low temperatures. The Co_3_O_4_ nanoparticles were supported on the surface of CeO_2_ nanorods, both of which crystallized well as seen from the clear lattice streaks in TEM images.

Wu et al. [124] uncovered a striking discovery that the precipitation of cobalt oxides in the presence of ceria using sodium or ammonium carbonate resulted in a highly heterogeneous sample mapping as observed by TEM and SEM analyses. The mapping displayed large agglomerations of CeO_2_ particles that were not directly interacting with the Co_3_O_4_ crystallites. Upon scrutinizing the morphology of the particles on its surface, they noticed that this trend was ubiquitous in all Co_3_O_4_-CeO_2_ mixed oxides.

**Figure 9:**
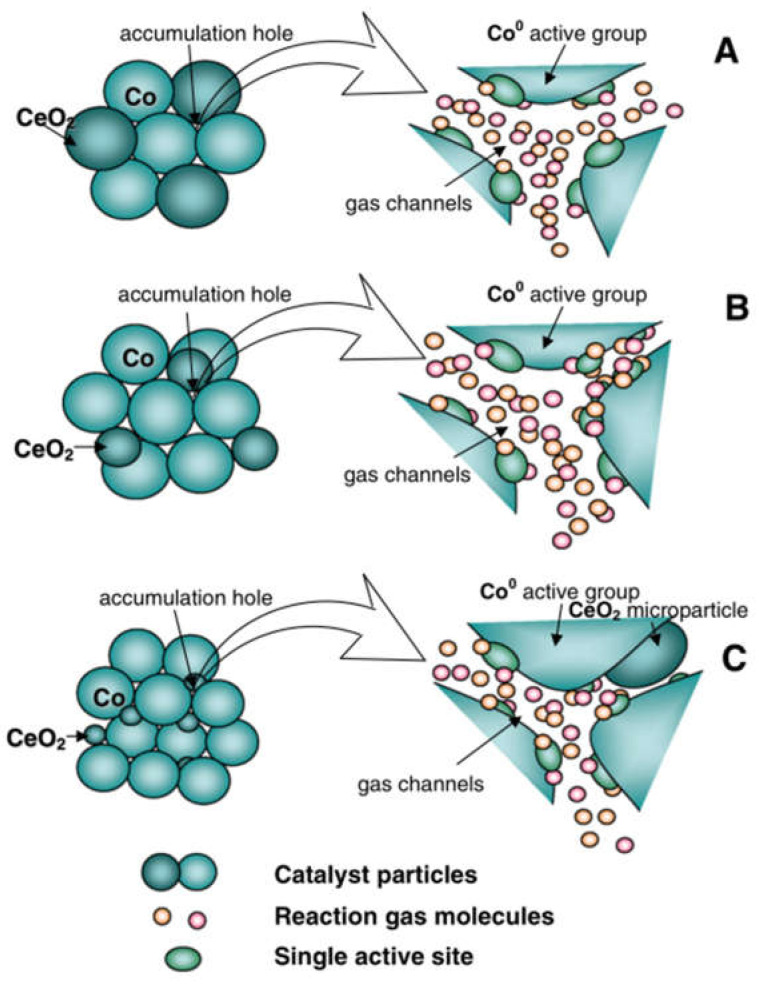
Schematic diagram of pore distribution over the CeO_2_/Co_3_O_4_ catalysts: (**A**) CeO_2_/Co_3_O_4_ (1:1 and 1:2); (**B**) CeO_2_/Co_3_O_4_ (1:3~1:6); (**C**) CeO_2_/Co_3_O_4_ (1:8). Reproduced with permission from S. Zeng, Influence of pore distribution on catalytic performance over inverse CeO_2_/Co_3_O_4_ catalysts for CH_4_/CO_2_ reforming; published by Fuel Processing Technology, 2013 [125].

Zeng et al. [125] have prepared CeO_2_/Co_3_O_4_ catalysts with two different pore sizes through hydrothermal and microemulsion methods. The catalysts with a Ce/Co molar ratio of 1:1 and 1:2 exhibit a single pore distribution as depicted in Figure 9A, while a two-pore distribution is observed for the catalysts with a Ce/Co molar ratio of 1:3~1:6 (Figure 9B). The micro- and mesopores in these catalysts provide ample active sites and channels for gas diffusion, resulting in enhanced catalytic activity for methane. However, when the Ce/Co molar ratio is further reduced to 1:8 (Figure 9C), the interaction between CeO_2_ and Co_3_O_4_ is weakened, leading to reduced catalytic activity. The total reduction degree of Co_3_O_4_ calculated by the separation peak is shown in Table 6. The reduction degree of Co_3_O_4_ was greater than 90% for the CeO_2_/Co_3_O_4_ (1:1) catalyst at a pretreatment temperature of 650 °C, indicating the availability of active sites for CO_2_ reforming. Ercolino [126] has synthesized iron cobalt spinel mixed catalysts by liquid phase combustion, doped with 3% Pd by wet impregnation. When iron atoms replaced 2/3 of cobalt atoms in the catalyst, the catalytic activity was found to be similar to that of the parent Co_3_O_4_.

Darda et al. [127] have conducted a comprehensive synthesis of various CeO_2_/Co_3_O_4_ catalysts using different synthesis routes. The resulting ceria carriers exhibited diverse physicochemical properties, which were further modified after Co incorporation. Interestingly, the hydrothermal synthesis route has been found to produce an improved CeO_2_ support with a smaller crystallite size, larger surface area, and enhanced reducibility, thereby establishing its superiority over other synthesis routes. It is noteworthy that the CeO_2_ support and the synthetic procedure played a critical role in terms of dispersing the active Co component and exhibiting higher oxygen mobility, as deduced by comparing the catalytic samples. The nanocomposite catalyst Co_3_O_4_/CeO_2_-H has emerged as the most promising candidate, displaying higher activity in the complete oxidation of CH_4_ compared to Co_3_O_4_/CeO_2_-P and pure CeO_2_ materials. The higher dispersion of the deposited Co species and the enhanced reducibility of Co-Ce catalysts advocate for synergistic effects of CeO_2_ nanorods and the supported Co_3_O_4_ nanoparticles, thereby establishing their critical role in the catalytic process. 

Choya et al. [128] synthesized six different Co_3_O_4_/CeO_2_ catalysts using the precipitation method and investigated their catalytic performance in the complete oxidation of methane. The researchers employed diverse synthesis routes to prepare CeO_2_ support and examined the effects of its physicochemical properties on the performance of the cobalt catalyst. Among the six synthesized CeO_2_ supports, those obtained by the CN (cerium nitrate hexahydrate) and DC (direct calcination) methodologies exhibited the best textural and structural properties, with specific surface areas of approximately 60 m^2^ g^−1^. The researchers evaluated the efficiency of the synthesized Co_3_O_4_/CeO_2_ catalyst by obtaining the light-off curve of the catalyst at 60,000 h^−1^, between 200 and 600 °C. Upon direct observation of the light-off curves of the catalyst, it was apparent that the light-off behavior of Co-CC (carbonate) catalyst was the worst, whereas the light-off efficiency of Co-DC catalyst was the best. Furthermore, through the analysis of the surface composition and redox performance of cerium support and cobalt catalyst, it was discovered that the content of Ce^4+^ ions supported by Co-DC and Co-CN catalysts on cerium support was significantly higher, meaning that the oxidation degree was higher. Consequently, both catalysts displayed a favorable presence of Co^3+^ ions on their surfaces, leading to higher concentrations of active lattice oxygen. For these reasons, these two catalysts were found to be the most active. Furthermore, due to the high hydrophobic properties of CeO_2_, the best catalyst in the combination (Co-DC) exhibited significant resistance to water vapor deactivation. These findings suggest that the synthesis method used to prepare CeO_2_ support plays a significant role in determining the physicochemical properties of the resulting catalyst, which, in turn, affects the catalytic performance.

**Figure 10:**
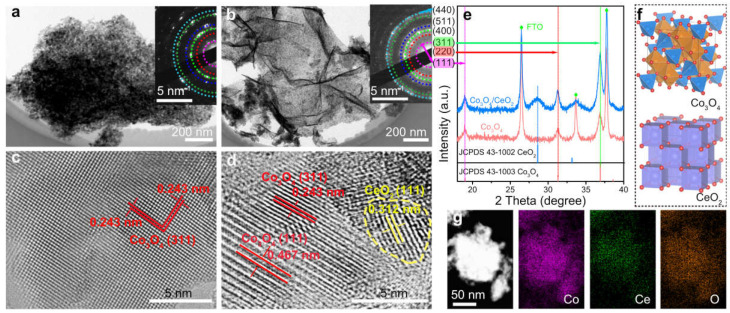
Structural characterizations of Co_3_O_4_ nanostructures and Co_3_O_4_/CeO_2_ nanocomposites. TEM images of (**a**) Co_3_O_4_ and (**b**) Co_3_O_4/_CeO_2_ nanosheets, the insets show the corresponding SAED patterns. HRTEM images of (**c**) Co_3_O_4_ and (**d**) Co_3_O_4_/CeO_2_ samples. The CeO_2_ domain is highlighted with a yellow dashed circle. (**e**) PXRD patterns of the samples on FTO substrates in comparison with the standard PXRD patterns of Co_3_O_4_ and CeO_2_. (**f**) Crystal structures of Co_3_O_4_ and CeO_2_. (**g**) Dark-field TEM image and the corresponding elemental mappings of Co, Ce, and O in the Co_3_O_4_/CeO_2_ sample. Reproduced with permission from Jinzhen Huang, Modifying redox properties and local bonding of Co_3_O_4_ by CeO_2_ enhances oxygen evolution catalysis in acid; published by nature [129].

Huang et al. [129] have just introduced a revolutionary method of enhancing the intrinsic catalytic activity of Co_3_O_4_ by forming heterogeneous Co_3_O_4_/CeO_2_ nanocomposites. Using nanocrystalline CeO_2_, they were able to establish Co_3_O_4_/CeO_2_ nanocomposites as active acidic OER catalysts. The process involved the direct synthesis of Co_3_O_4_ nanostructures and Co_3_O_4_/CeO_2_ nanocomposites on fluorine-doped tin oxide (FTO) electrodes by electrodepositing corresponding metal hydroxide precursors, which were then annealed in air. The HRTEM images (Figure 10) revealed nanocrystalline domains in Co_3_O_4_ and Co_3_O_4_/CeO_2_ samples, indicating a reduction in crystallinity due to the introduction of CeO_2_. The two samples showed polycrystalline properties, with electron diffraction patterns of selected regions showing similar diffraction rings. The inner and outer diffraction rings were labeled as the (111), (220), (311), (400), (511), and (440) planes of Co_3_O_4_, consistent with the PXRD pattern and crystal structure of Co_3_O_4_ spinel oxide. The introduction of Co_3_O_4_/CeO_2_ reduced the average crystallization domain size of Co_3_O_4_ and the width of (311) diffraction peak estimated by Co_3_O_4_ /CeO_2_, indicating the successful introduction of Ce into the nanocomposite. The team found that the introduction of CeO_2_ inhibited the pre-OER redox characteristics of Co_3_O_4_ in the acidic medium, indicating the instability of the dimerized CoIV intermediate. The introduction of CeO_2_ also dispersed phase pure CeO_2_ nanocrystals between phase pure Co_3_O_4_ crystals, greatly inhibiting the pre-OER redox characteristics of Co_3_O_4_ in the acidic medium. This novel method paves the way for the development of more efficient and effective OER catalysts, with a wide range of potential applications.

Researchers found that the highly dispersed Co_3_O_4_ particles promoted by ZrO_2_ have been claimed as active sites for methane oxidation with low cobalt content. Kim, Jiwon et al. [130] have uncovered that ZrO_2_ nanotube powder, endowed with a stupendously high specific surface area, has been adorned with Co_3_O_4_ nanoparticles, and utilized as an electrochemical anode for the partial oxidation of methane, ultimately leading to the generation of C_3_ alcohol products. Impressively, the highly dispersed Co_3_O_4_ particles, propelled by ZrO_2_, have been heralded as active sites for methane oxidation, despite the low cobalt content. The Co_3_O_4_ nanoparticles, decorated on the outer surface of the ZrO_2_ nanotubes, offer a highly accessible diffusion route for methane gas, culminating in a low onset potential for electrochemical methane activation. This ground-breaking discovery has culminated in the achievement of the long-sought-after objective of electrochemical catalytic methane combustion.

**Figure 11:**
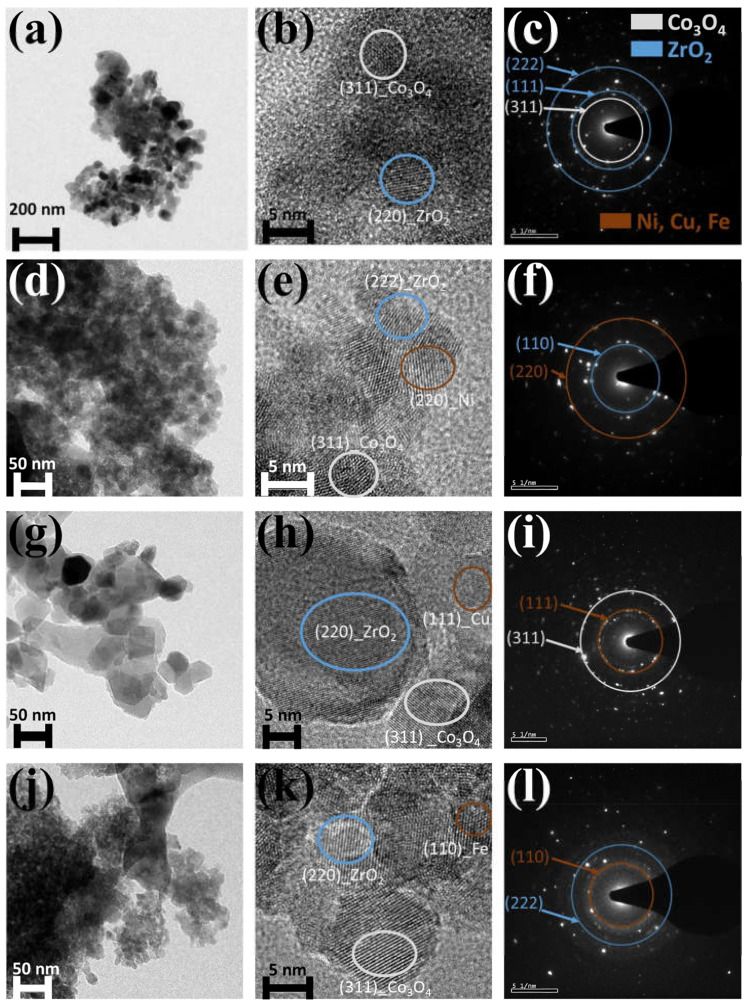
Bright field, high resolution, and selective area diffraction images of (**a**–**c**) Co_3_O_4_-ZrO_2_, (**d**–**f**) Ni/Co_3_O_4_-ZrO_2_, (**g**–**i**) Cu/Co_3_O_4_-ZrO_2_, and (**j**–**l**) Fe/Co_3_O_4_-ZrO_2_. Reproduced with permission from Satyapaul A. Singh, Transition Metal (Ni, Cu and Fe) Substituted Co_3_O_4_-ZrO (2) Catalysts for Lean Methane Combustion; published by Topics in Catalysis 2021 [37].

Singh et al. [37] prepared transition metal (Ni, Cu, and Fe) substituted Co_3_O_4_-ZrO_2_ catalysts by PEG assisted Sono chemical synthesis. In Figure 11, the HRTEM images and selective area diffraction patterns of Co_3_O_4_-ZrO_2_ (a–c), Ni/Co_3_O_4_-ZrO_2_ (d–f), Cu/Co_3_O_4_-ZrO_2_ (h–j), and Fe/Co_3_O_4_-ZrO_2_ (k–l) show that Ni, Cu, and Fe substituted well. Lean methane combustion was tested with prepared catalysts. The results show that T_90_ of Co_3_O_4_-ZrO_2_, Ni/Co_3_O_4_-ZrO_2_, Cu/Co_3_O_4_-ZrO_2_, and Fe/Co_3_O_4_-ZrO_2_ catalysts are 538 °C, 533 °C, 518 °C, and 511 °C.

Co_3_O_4_ supported on SmMn_2_O_5_ composite catalysts synthesized by deposition–precipitation method by Feng et al. [131] took a gander at Co_3_O_4_ supported on SmMn_2_O_5_ composite catalysts that were synthesized using the deposition–precipitation method. These catalysts were put to the test for methane catalytic combustion in an oxygen-rich atmosphere. After Co loading, the methane combustion catalytic activity was significantly boosted, and SmMn_2_O_5_ proved to be the key player in stabilizing the Co_3_O_4_ against sintering. This kind of mutual promotion paid off, with the Co/SMO-50% (50% represents the proportion of SmMn_2_O_5_) catalyst displaying overall superior catalytic performance with high catalytic activity and strong durability. Highly dispersed small Co_3_O_4_ nanoparticles induced the formation of more surface-adsorbed oxygen and improved the reducibility, while the activity of surface lattice oxygen also got a boost. Additionally, the thermally stable SmMn_2_O_5_ partially prevented the aggregation of Co_3_O_4_, resulting in low activity loss under recycle and long-term tests. As shown in Table 7, the content, reaction conditions, and temperature of several Co_3_O_4_ composites catalysts doped with noble metals or metal oxides in methane combustion are listed.

In conclusion, the appropriate amount of metal oxide doping can enhance the oxygen activity and stability of Co–O bond and elevate the concentration of active oxygen [132,133,134,135], thus improving the catalytic efficiency of methane oxidation reaction [102,136,137,138]. For example, doping of CeO_2_ can introduce a large number of oxygen vacancies into the Co_3_O_4_ lattice, forming more oxygen active sites on the Co–O bond, thereby increasing the oxygen reduction capacity of the Co_3_O_4_ catalyst surface and the adsorption capacity of methane molecules. In addition, doping of CeO_2_ can improve the interaction force and coordination environment between Co and O ions in Co_3_O_4_ catalyst, and promote the formation and stability of Co–O bond. Doping of ZrO_2_ can change the chemical properties of the surface of Co_3_O_4_ (acid–alkalinity, oxidation–reduction), thus enhancing the strength of Co–O. In addition, ZrO_2_ can also cause lattice distortion, increase surface defects, promote the formation of Co–O bonds, and improve its oxygen activity and stability. Additionally, the other dopants have similar effects.

### 3.3. Non Metallic Oxides

The doping of nonmetallic oxides can affect the bond length and bond strength of Co–O bond, and then affect the activity, selectivity, and finally regulate methane catalysis. SiO_2_ can form Co–O–Si bonds with Co_3_O_4_, which have a longer bond length than Co–O bonds, and the doping of SiO_2_ will also increase the specific surface area of Co_3_O_4_. All these changes lead to increased reducibility of Co_3_O_4_ and promote the oxidation of methane. It is worth noting that the SiO_2_ carrier is completely inert to the Co_3_O_4_ active phase reaction, whereas silica possesses a host of benefits, including exceptional resistance to acidic substances, high-temperature endurance, robust wear resistance, and commendable mechanical strength [83,139,140,141,142,143].

**Figure 12:**
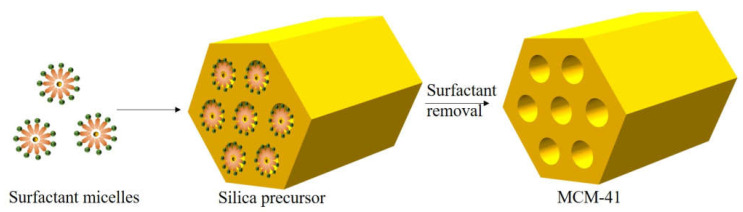
The schematic illustration of MCM-41.

In the 1990s, Kresge et al. [144] reported a surprising discovery, where they introduced a groundbreaking mesoporous crystalline material that was named MCM-41. It was a new genre of nano-structured material that boasted the properties of being hexagonally arranged in an orderly manner, along with a uniform size. The pore size of this marvel could be adjusted continuously in the range of 2–10 nm, with an added advantage of a large specific surface area. It is important to note that the key component of the MCM-41 [145,146,147,148,149] molecular sieve is amorphous silica. It is noteworthy that mesoporous silica is comparatively richer, which facilitates the mass transfer process. Furthermore, the sizeable specific surface area allows high concentrations of active substances to be swiftly transmitted on the surface [150]. The MCM-41 molecular sieve plays a crucial role in the dispersal and isolation of the active phase of the catalyst in the composite catalyst. The MCM-41 molecular sieve has a considerable specific surface area. Its adsorption quantity is relatively copious, and the pore size distribution has a relatively large uniformity and regulation. Its stable chemical properties make it a hot spot for research [151]. The amalgamation of subgroups and VIII elements with molecular sieves has high catalytic activity and selectivity in the oxidation of organic compounds [152], thereby making it a trending topic of research by replacing silicon atoms in molecular sieves. The substantial specific surface area and homogeneous skeleton structure of the MCM-41 molecular sieve can be doped with metal atoms. These atoms can be doped at the defect site of the molecular sieve to form a skeleton counterion or additional skeleton oxides [153]. Figure 12 showcases the ideal hexagonal structure of the MCM-41 molecular sieve, further elucidating its outstanding characteristics.

In the field of catalysis, the preparation of composite catalysts with high dispersion of active components has become a hot topic of research. In this regard, Sangyun Lim et al. [154] used a hydrothermal method to directly mix a cobalt-containing precursor solution with MCM-41 molecular sieve, resulting in the formation of a composite catalyst with high dispersion of cobalt. The presence of Co in the composite catalyst was confirmed using ultraviolet-visible spectroscopy. Furthermore, through the use of X-ray absorption fine structure (XAFS) detection, it was found that cobalt atoms were uniformly distributed in the pores of molecular sieves in the form of near atomic dispersion. Pandurangan [155] synthesized a series of mesoporous Co-MCM-41 molecular sieves with different Si/Co ratios by directly introducing cobalt into the skeleton by hydrothermal method. After ultraviolet spectroscopy analysis, it was discovered that the sample contained Co^2+^ and Co^3+^, and some Co^2+^ were attached to the skeleton of the molecular sieve. This attachment not only enhances the stability of the molecular sieve, but also provides more opportunities for the cobalt atoms to be dispersed uniformly throughout the pores of the molecular sieve.

The interplay between pore size and catalyst performance is a critical factor to consider. In the study conducted by Panpranot et al. [156], a hydrothermal method was employed to repurpose the original MCM-41 molecular sieve (M1) into a molecular sieve with a larger pore size (M2). The data analysis using SEM in combination with EDX revealed that the M1 molecular sieve possesses a relatively small pore size. Co ions are predominantly distributed at the edges and surfaces of M1, while M2 is associated with amorphous SiO_2_. The particle size of both molecular sieves is relatively large, but Co is more uniformly dispersed in M2 than in M1. Compared to amorphous SiO_2_, the smaller pore size of MCM-41 zeolite results in an increased intensity of the interaction between Co and the carrier, leading to the formation of more stable Co_3_O_4_ that is less likely to be inactivated during the actual catalytic process.

**Figure 13:**
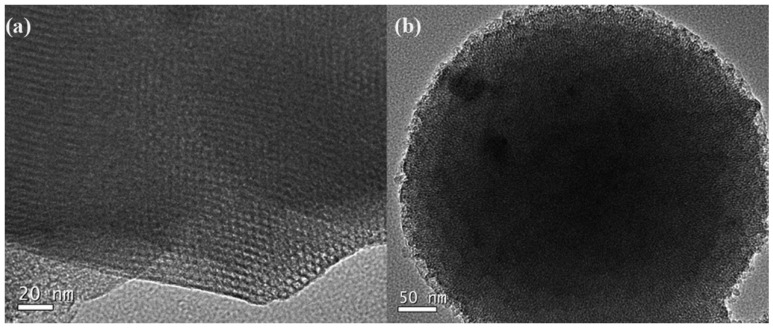
TEM images of (**a**) 60Co-MCM-41sample; (**b**) 60Co-MCM-41imp sample. Reproduced with permission from X. Xu, High-efficiency non-thermal plasma-catalysis of cobalt incorporated mesoporous MCM-41 for toluene removal; published by Catalysis Today, 2016 [157].

The dispersion of cobalt in composite catalysts can vary depending on the preparation method used. When cobalt ions are added through impregnation, they tend to cluster together on the outer surface of the MCM-41 molecular sieve in the form of cobalt oxide clusters or Co_3_O_4_ nanoparticles. However, when the catalyst is prepared using the hydrothermal method, cobalt ions are more uniformly distributed in tetrahedral coordination sites [157]. The catalytic activity sequence for different preparation methods is hydrothermal > co-precipitation > impregnation. Xu et al. [157] used sodium silicate as the silicon source and synthesized the composite catalyst using the direct hydrothermal method with cobalt modification. The resulting MCM-41 molecular sieve retained its mesoporous structure, as shown in Figure 13, with almost no nanoparticles or metal oxide clusters present outside the mesoporous channels. This indicates that cobalt is doped into the Si-O skeleton of Co-MCM-41, resulting in a clear and orderly mesoporous structure. In contrast, small CoO_x_ clusters or particles were observed on the surface of the Co-MCM-41 imp sample, as shown in Figure 13b. Atomic absorption spectroscopy and gas chromatography-mass spectrometry data revealed that Co-MCM-41 exhibits good dispersion of cobalt elements in the catalyst, leading to enhanced stability of the catalyst.

Todorova et al. [158] utilized the hydrothermal method to introduce vanadium (V) elements into the precursor solution, thereby synthesizing a mesoporous molecular sieve of cobalt-based MCM-41 replete with V ions. The incorporation of V ions, as revealed by their study, effectively mitigated the interaction of cobalt oxides with MCM-41 and concomitantly amplified the Co^3+^ content, thereby endowing it with superior catalytic potential.

**Figure 14:**
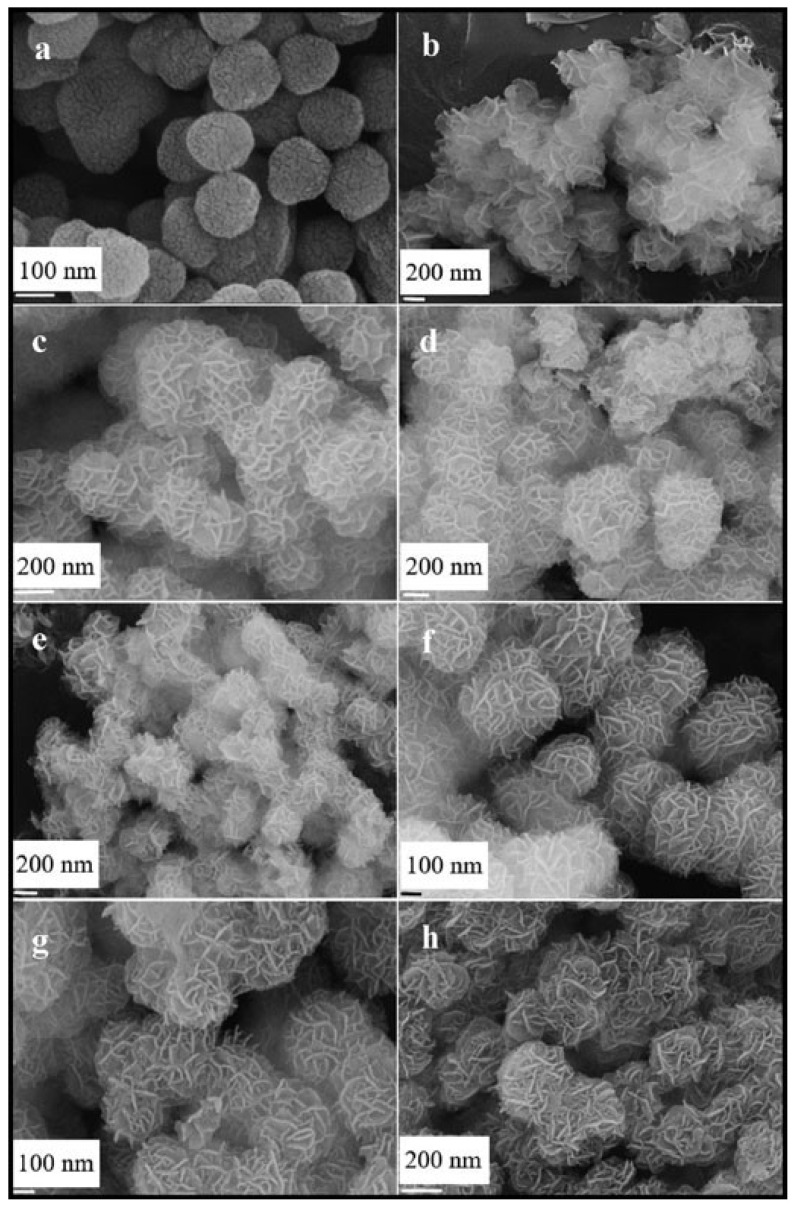
SEM images of different samples. (**a**) SiO_2_, (**b**) Co_3_O_4/_SiO_2_, (**c**) Mn_0.4_/Co/SiO_2_, (**d**) Mn_0.6_/Co/SiO_2_, (**e**) Mn_0.8_/Co/SiO_2_, (**f**) Mn_1_/Co/SiO_2_, (**g**) Mn_2_/Co/SiO_2_, and (**h**) Mn_4_/Co/SiO_2_. Reproduced with permission from Haiwang Wang, Enhancing catalytic CH_4_ oxidation over Co_3_O_4_/SiO_2_ core-shell catalyst by substituting Co^2+^ with Mn^2+^; published by Journal of Dispersion Science and Technology 2021 [159].

Undoubtedly, SiO_2_/Co_3_O_4_ exhibits remarkable catalytic performance; however, as the recent research by Haiwang Wang et al. [159] indicates, replacing cobalt with other metal elements could significantly improve its efficacy. By employing the hydrothermal method, they synthesized a core-shell flower-spheroidal composite catalyst comprising cobalt and manganese, which aptly leveraged the carrier’s advantages, special morphology, and doping modification to curtail the risk of sintering and deactivation of Co_3_O_4_ and demonstrated superior catalytic activity. In comparison with Co_3_O_4_/SiO_2_, methane conversion at 350 °C and 450 °C was observed to augment by 10% and 6%, respectively. Figure 14 underscores the effect of varying manganese oxide doping amounts on the catalyst’s microstructure. Remarkably, Mn_X_/Co/SiO_2_ catalyst exhibited a greater dispersion than Co_3_O_4_/SiO_2_ catalyst, with the nanosheet’s arrangement being more orderly. Thus, manganese ions appear to have a twofold benefit: first, they optimize the microstructure of Co_3_O_4_, and second, they improve its dispersibility, thereby elevating its catalytic performance.

## 4. Summary and Outlook

This manuscript presents a complex and intricate overview of the catalytic oxidation mechanism of nano-Co_3_O_4_, followed by an extensive survey of the recent progress made in the development of Co_3_O_4_ composite catalysts containing metal elements, metal oxides, and non-metallic oxides for methane combustion. The paper also discusses the challenges and conveniences involved in using various Co_3_O_4_ composite catalysts and highlights the measures that can be taken to enhance their catalytic performance.

The paper presents three measures to enhance the catalytic performance of Co_3_O_4_ catalysts, namely (1) doping with noble metal elements or compounding with metal oxides and non-metal oxides, (2) regulating the microscopic morphology of the composite catalysts, and (3) improving the preparation process. The researchers have found that the synergistic effect between the components of the Co_3_O_4_ composite catalysts significantly improves their catalytic performance. By doping noble metals into Co_3_O_4_, the noble metal atoms affect the crystal structure of Co_3_O_4_, and the doping of noble metals weakens the Co–O bond and promotes the formation of active oxygen, thus improving its catalytic effect. By compounding Co_3_O_4_ with other metal oxides, the crystal structure of Co_3_O_4_ was significantly changed, with more dense catalytic sites and higher catalytic activity, resulting in improved performance. By loading Co_3_O_4_ on non-metallic oxides, its own crystal structure changes less. Because of the dispersion of Co_3_O_4_ on the surface of non-metallic oxides, its surface area increases and it is more difficult to sinter, which makes the catalytic performance improved. The influence of three types of dopants on Co–O in Co_3_O_4_ catalytic materials is reflected in the following aspects: (1) doping of noble metals can improve the activity and stability of the catalyst (by introducing more oxygen vacancies, increasing the content of Co–O bond on the surface of Co_3_O_4_), and can also affect the fracture and formation of Co–O bond, promoting the formation and release of Co_3_O_4_ surface oxygen in methane oxidation reaction; (2) doping of metal oxides can improve the oxygen reduction ability of Co_3_O_4_ catalyst surface (by introducing oxygen vacancy, more oxygen active sites are formed on the Co–O bond); and (3) doping of non-metallic oxides will affect the bond length and bond strength of Co–O bonds, and then affect its activity and selectivity.

Furthermore, by designing the microscopic morphology of the composite catalysts, researchers can produce a large number of mesoporous structures that increase the specific surface area and improve the catalytic performance. In addition, different preparation methods have their own advantages. For example, the hydrothermal method, which mainly adopts low-temperature liquid-phase control, is a simple process and easy to adjust the reaction process. It does not require high-temperature treatment, and the obtained catalyst products have completed crystalline shape, uniform particle size distribution, and good dispersion.

In addition, catalytic stability is divided into chemical stability, heat stability, anti-toxicity stability, and mechanical stability. Chemical stability refers to the maintenance of the chemical composition and chemical state of the catalyst during the catalytic process. Thermal stability refers to the ability of a catalyst to maintain catalytic activity under harsh conditions such as high temperature. Catalysts are more unstable under high temperatures and are prone to sintering and agglomeration, which reduces the surface area and thus affects the catalytic performance. However, the micro-porous structure and other doping elements introduced in this paper can not only change the catalytic ability, but also reduce the effect of sintering and agglomeration to some extent, thereby improving the thermal stability.

It should also be noted that studies indicate that there may be a shortage of cobalt supply in 2050. Effective ways to solve this problem are as follows: (1) finding alternative materials such as Ni, Mn, Zn, and other transition group metals with good properties; (2) reducing the amount of cobalt without reducing the catalytic performance, which means improving the atomic efficiency of cobalt; and (3) further developing more efficient, convenient, and low-cost technologies for Co_3_O_4_ catalyst recovery.

## Figures and Tables

**Table 1:** Specific surface area and corresponding preparation temperature of several nano Co_3_O_4_.

Catalyst	Temperature (°C)	BET (m^2^ g^−1^)	Reference
Nanoplate Co_3_O_4_	325	45.5	[47]
Nanoparticle Co_3_O_4_	150	15	[45]
	325	112.6	[47]
	550	46	[45]
	650	42	[45]
Nanorob Co_3_O_4_	325	111.4	[47]
	90	170.2	[48]
Nanotube Co_3_O_4_	350	36–48	[48]
Bulk Co_3_O_4_	700	20.9	[48]
Mn promoted Co_3_O_4_ spinel (Cat-R)	340	127.94	[50]
Mn promoted Co_3_O_4_ spinel (Cat-S)	420	57.43	[50]
Mn promoted Co_3_O_4_ spinel (Cat-F)	380	94.5	[50]

**Table 2:** The mechanism of three measures to enhance the catalytic performance of Co_3_O_4_ catalyst for methane oxidation catalyzed by Co_3_O_4_.

Measures	Results	Specific Mechanism
pure Co_3_O_4_		The catalytic performance of pure Co_3_O_4_ is poor due to the lack of support and synergy of these vectors.
doping with noble metal elements	The specific surface area of Co_3_O_4_ is increased, and the physical and chemical properties of the surface are changed, to improve the catalytic performance.	By providing additional protons and electrons in the catalytic reaction, the reducing power and catalytic activity of Co_3_O_4_ are increased, while also facilitating the contact between the cobalt tetroxide and the reactants, thus improving the efficiency of the catalyst.
compounding with metal oxides		By helping Co_3_O_4_ to form more active sites on the surface, the rate of catalytic reaction is increased.
compounding with non-metal oxides		By helping Co_3_O_4_ to disperse evenly on the surface of the carrier, the contact area of the catalytic reaction is increased and the catalytic efficiency is improved.

**Table 3:** Physical properties and CH_4_ catalytic activities of the as-prepared catalysts. Reproduced with permission from Xiong, The effect of existence states of PdO_x_ supported by Co_3_O_4_ nanoplatelets on catalytic oxidation of methane; published by Applied Surface Science, 2021 [82].

Catalyst	SBET (m^2^ g^−1^) ^a^	Co_3_O_4_ Particle Size (nm) ^b^	T_10_ (°C) ^d^	T_50_ (°C) ^d^	T_90_ (°C) ^d^	Ea (kJ mol^−1^) ^e^
Co_3_O_4_	25.6	13.4	298	357	402	138.0
Pd-Co_3_O_4_	28.7/26.9 ^c^	13.2	247	291	337	66.9
Pd/Co_3_O_4_	21.6	13.8	265	315	361	89.7
Pd@Co_3_O_4_	23.6	14.5	280	326	372	90.5

^a^ From N_2_ adsorption–desorption results. ^b^ The Co_3_O_4_ particle size is calculated by Scherrer equation based on the diffraction peak broadening. ^c^ The surface areas of 26.9 m^2^ g^−1^ shows the spent Pd-Co_3_O_4_ catalysts after H_2_O and CO_2_-resistance test. ^d^ The reaction was carried out in a feed gas of 1% CH_4_, 20% O_2_, gas hourly space velocity: 30,000 mL g^−1^ h^−1^. ^e^ The apparent activation energy (Ea) was tested by tuning the temperature between 210 and 240 °C.

**Table 4:** XPS data of Pd/Co_3_O_4_ and Co_3_O_4_ as prepared and after catalytic tests. The uncertainty on the atomic concentration is of the order of 10%. Reproduced with permission from Liotta, Pd/Co_3_O_4_ catalyst for CH_4_ emissions abatement: study of SO_2_ poisoning effect; published by Topics in Catalysis, 2007 [99].

Sample	Co 2p_3/2_ (eV)	S 2p (eV)	Co (at%)	Pd (at%)	S (at%)
Pd/Co_3_O_4_	779.6		38.8	4.0	
	781.4				
Pd/Co_3_O_4_ after 4 runs without SO_2_	779.5		36.3	0.4	
	780.8				
Pd/Co_3_O_4_ after 4 runs with SO_2_ (1, 10 ppm)	779.9	169.5	34.8	0.2	1.9
	781.3				
Pd/Co_3_O_4_ after 15 h at 350 °C with 10 ppm SO_2_ and 1 run without SO_2_	780.0	169.5	33.5	0.4	2.4
	781.4				
Co_3_O_4_	779.5		38		
	780.0				
Co_3_O_4_ after 15 h at 350 °C with 10 ppm SO_2_ and 1 run without SO_2_	779.9	169.6	36.6		5.0
	781.1				
Co_3_O_4_ after 15 h at 350 °C with 10 ppm SO_2_ and 3 runs without SO_2_	779.7	169.6	37.5		1.1
	781.1				

**Table 5:** Au and Pd weight loading of the samples calculated from ICP-AES, CO chemical adsorption amounts (CAA), adsorbed oxygen/lattice oxygen molar ratio (O_ads_/O_latt_), Pd^x+^/Pd^0^ molar ratio (x ≥ 2), reaction rate (r) (calculated by weight amount of the catalysts), apparent activation energy (Ea), and the temperatures for 10% and 90% conversion of methane (T_10_ and T_90_). Reproduced with permission from Yang, Au@PdO_x_ with a PdO_x_-rich shell and Au-rich core embedded in Co_3_O_4_ nanorods for catalytic combustion of methane; published by Nanoscale, 2017 [106].

Catalysts	Pd Content (wt.%)	Au Content (wt.%)	CO CAA (μmol g^−1^)	Pd^x+^/Pd^0^ Molar Ratio	O_ads_/O_latt_ Molar Ratio	r (μmol g^−1^ s^−1^)	Ea (kJ mol^−1^)	T_10_ (°C)	T_90_ (°C)
Au@PdO_x_ (1:5)/Co_3_O_4_	2.44	0.49	92.8	29.3	0.51	194	50.9	210	344
AuPd (1:5)/Co_3_O_4_	2.44	0.49	65.1	21.2	0.45	101	72.2	237	350
Pd/Co_3_O_4_	2.45	0	123.6	26.7	0.49	70	63.0	251	372
Au/Co_3_O_4_	0	2.70	57.2	-	0.44	75	53.5	250	>450
Co_3_O_4_	0	0	114.8	-	0.48	25	74.0	281	500

**Table 6:** The coke deposition of the CeO_2_/Co_3_O_4_ catalysts. Reproduced with permission from S. Zeng, Influence of pore distribution on catalytic performance over inverse CeO_2_/Co_3_O_4_ catalysts for CH_4_/CO_2_ reforming; published by Fuel Processing Technology, 2013 [125].

Catalyst	Total Reduction Degree of Co_3_O_4_	Reaction Temp and Time	Coke Deposition (wt.%)
CeO_2_/Co_3_O_4_ (1:1)	0.8938	750 °C, 5 h	40.84
CeO_2_/Co_3_O_4_ (1:2)	0.9073	750 °C, 5 h	53.11
CeO_2_/Co_3_O_4_ (1:3)	0.9662	750 °C, 5 h	61.21
CeO_2_/Co_3_O_4_ (1:4)	0.9743	750 °C, 5 h	61.04
CeO_2_/Co_3_O_4_ (1:5)	0.9350	750 °C, 5 h	68.62
CeO_2_/Co_3_O_4_ (1:6)	0.9159	750 °C, 5 h	61.52
CeO_2_/Co_3_O_4_ (1:8)	0.9526	750 °C, 5 h	47.33

**Table 7:** The content, reaction conditions, and temperature of several Co_3_O_4_ composites catalysts doped with noble metals or metal oxides in methane combustion.

Catalysts	Content	Reaction Conditions	Temperature				Reference
			T_10_ (°C)	T_50_ (°C)	T_90_ (°C)	T_100_ (°C)	
Co_3_O_4_		1 vol.% CH_4_, 4 vol.% O_2_, WHSV 78,000 mL g^−1^ h^−1^		597			[37]
Pd/Co_3_O_4_	3 wt.% Pd coated with SiC OCF	0.5 vol.% CH_4_, 30 WHSV NL s^−1^ g^−1^_cat_	272	305	350		[93]
Pd/Co_3_O_4_	3 wt.% Pd coated with Zir OCF	0.5 vol.% CH_4_, 30 WHSV NL s^−1^ g^−1^_cat_	220	250	275		[93]
Pd/Co_3_O_4_	0.7 wt.% Pd	0.3 vol.% CH_4_, 0.6 vol.% O_2_/He	300–350	383	500–550		[99]
Au/Co_3_O_4_	0.18 wt.% Au	1 vol.% CH_4_, 5 vol.% O_2_, and N_2_ balance	241	317	370		[100]
Pt/Co_3_O_4_	0.21 wt.% Pt	2 vol.% CH_4_, 5 vol.% O_2_, and N_2_ balance	238	312	358		[100]
Au-Pt/Co_3_O_4_	1.92 wt.% Au and 1.63 wt.% Pt	3 vol.% CH_4_, 5 vol.% O_2_, and N_2_ balance	218	295	332		[100]
Au-Pd/Co_3_O_4_	1.90 wt.% Au and 1.48 wt.% Pd	4 vol.% CH_4_, 5 vol.% O_2_, and N_2_ balance	241	317	363		[100]
Co_3_O_4_/SnO_2_	Co/(Co + Sn) = 0.75	1.0 vol.% CH_4_, 10.0 vol.% O_2_, and N_2_ balance; GHSV 18,000 mL g_cat_^−1^ h^−1^				753	[49]
Co_3_O_4_/γ-Al_2_O_3_	30 wt.% Co_3_O_4_	0.2 vol.% CH_4_, 10 vol.% O_2_, and N_2_ balance; GHSV 36,000 mL h^−1^ g^−1^	300			550	[107]
Co_3_O_4_/γ-Al_2_O_3_	10.0 wt.% Co_3_O_4_	1.0 vol.% CH_4,_ space velocity 15,000 h^−1^		320–340		400	[59]
Co_3_O_4_/CeO_2_		20 mL min^−1^ of 10% CH_4_/Ar and 10 mL min^−1^ of O_2_ WHSV of 9000 mL·g^−1^·h^−1^			450		[123]
Co_3_O_4_/CeO_2_	30 wt.% Co_3_O_4_	0.3 vol.% CH_4_, 0.6 vol.% O_2_, and He balance WHSV 60,000 mL g^−1^ h^−1^		451	549		[124]
Pd/Co_3−x_Fe_x_O_4_	3 wt.% Pd, x = 1.1	0.5 vol.% CH_4_		481			[126]
Co_3_O_4_/CeO_2_	15 wt.% Co	0.5% vol. CH_4_ and 10% vol. O_2_, He balance		440			[127]
SmMn_2_O_5_/Co_3_O_4_	Co/SMO-50%	1 vol.% CH_4_, 10 vol.% O_2_, N_2_ balance, and WHSV 60,000 mL g^−1^ h^−1^	334	390	437		[131]

## Data Availability

Not applicable.
